# Neurovascular Inflammaging in Health and Disease

**DOI:** 10.3390/cells9071614

**Published:** 2020-07-04

**Authors:** Ádám Mészáros, Kinga Molnár, Bernát Nógrádi, Zsófia Hernádi, Ádám Nyúl-Tóth, Imola Wilhelm, István A. Krizbai

**Affiliations:** 1 Institute of Biophysics, Biological Research Centre, 6726 Szeged, Hungary; meszaros.adam@brc.hu (Á.M.); molnar.kinga@brc.hu (K.M.); bernatnogradi@gmail.com (B.N.); zsofia.hernadi@gmail.com (Z.H.); nyul-toth.adam@brc.hu (Á.N.-T.); wilhelm.imola@brc.hu (I.W.); 2 Doctoral School of Biology, University of Szeged, 6726 Szeged, Hungary; 3 Theoretical Medicine Doctoral School, University of Szeged, 6720 Szeged, Hungary; 4 Foundation for the Future of Biomedical Sciences in Szeged, Szeged Scientists Academy, 6720 Szeged, Hungary; 5 Vascular Cognitive Impairment and Neurodegeneration Program, Reynolds Oklahoma Center on Aging/Oklahoma Center for Geroscience, Department of Biochemistry and Molecular Biology, University of Oklahoma Health Sciences Center, Oklahoma City, OK 73104, USA; 6 Institute of Life Sciences, Vasile Goldiş Western University of Arad, 310414 Arad, Romania

**Keywords:** aging, inflammaging, inflammasome, neurodegeneration, neuroinflammation, neurovascular unit, stroke

## Abstract

Aging is characterized by a chronic low-grade sterile inflammation dubbed as inflammaging, which in part originates from accumulating cellular debris. These, acting as danger signals with many intrinsic factors such as cytokines, are sensed by a network of pattern recognition receptors and other cognate receptors, leading to the activation of inflammasomes. Due to the inflammasome activity-dependent increase in the levels of pro-inflammatory interleukins (IL-1β, IL-18), inflammation is initiated, resulting in tissue injury in various organs, the brain and the spinal cord included. Similarly, in age-related diseases of the central nervous system (CNS), inflammasome activation is a prominent moment, in which cells of the neurovascular unit occupy a significant position. In this review, we discuss the inflammatory changes in normal aging and summarize the current knowledge on the role of inflammasomes and contributing mechanisms in common CNS diseases, namely Alzheimer’s disease, Parkinson’s disease, amyotrophic lateral sclerosis and stroke, all of which occur more frequently with aging.

## 1. Introduction

Almost without exception [1,2], aging is an inevitable, natural biological process for all organisms from invertebrates to mammals, characterized by a progressive and generalized functional impairment [3]. Being associated with physical and mental decline, aging has always been and continues to be a grave concern for humans [3]. According to a global population aging report released by the United Nations last year (https://www.who.int/ageing/en/), the number of people aged 65 years or over is expected to double in the next 30 years, reaching 1.5 billion people. Furthermore, the percentage of the aged population rose from 6% in 1990 to 9% in 2019 all over the world, and the rate is estimated to increase to 16% by 2050 [4]. Although a continuous increase in life expectancy and extension of longevity are indeed reflecting the essential success of medical, economic and social development, the other side of the coin cannot be ignored. This drastic population aging will have substantial and long-lasting social, economic and public health costs and consequences. Aging is the major risk factor for countless chronic diseases, which challenge both researchers and health professionals [5,6]. Also at the individual level, elderly people normally have to face the hardship of frailty that is associated with some degree of cognitive decline [7]. Overall, understanding the biogerontological process is therefore vital in health and disease.

Various complementary theories have been formulated about the mechanisms of aging, of which inflammaging, a concept proposed by Franceschi et al. [8], has been embraced by many researchers [9,10,11,12,13]. Inflammaging represents a persistent low-grade systemic inflammation with inapparent clinical symptoms. In fact, it operates as a seesaw with a progressive pro-inflammatory “overload”. Cytokines, such as interleukins (IL-1β, IL-6, IL-18, etc.) and tumor necrosis factor α (TNFα), as well as a gamut of self-debris (free radicals, extracellular ATP, high mobility group box-1 (HMGB1), urate crystals, ceramides, cardiolipin, succinate, palmitate, lipofuscin, β-amyloids, tau protein aggregates, α-synuclein fibrils, mitochondrial and nuclear DNA) originated from dysfunctional cells fuel the constant activated state of the immune system [14,15]. With aging, accumulation of these endogenous signals is less compensated by the autophagic machinery [15].

**Figure 1:**
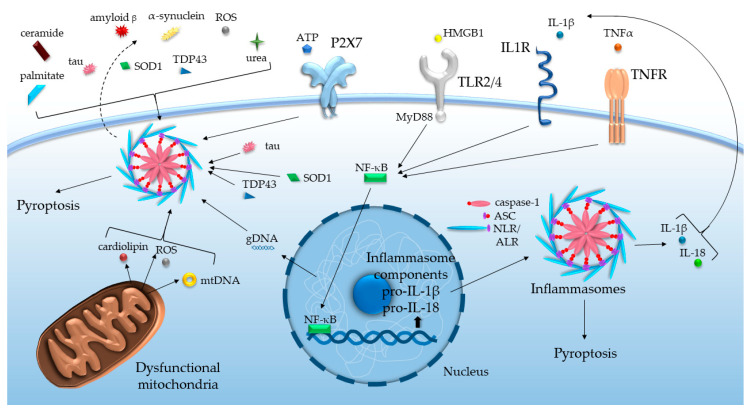
Inflammasome activation by various age-related DAMPs and cytokines. ALR: absent in melanoma 2-like receptor, ASC: apoptosis-associated speck-like protein containing a caspase recruitment domain, ATP: adenosine triphosphate, gDNA/mtDNA: genomic/mitochondrial deoxyribonucleic acid, HMGB1: high mobility group box-1, IL: interleukin, IL1R: interleukin-1 receptor, MyD88: Myeloid differentiation primary response 88, NF-κB: nuclear factor kappa-light-chain-enhancer of activated B cells, NLR: nucleotide-binding oligomerization domain-like receptor, ROS: reactive oxygen species, SOD1: superoxide dismutase 1, TDP43: TAR DNA-binding protein 43, TLR: Toll-like receptor, TNF(R): tumor necrosis factor (receptor).

The stressors listed above function as damage-associated molecular patterns (DAMPs), activating the pattern recognition receptors (PRRs) of the innate immune system. Notably, among PRRs, Toll-like receptors (TLRs), nucleotide-binding oligomerization domain-like receptors (NLRs) and absent in melanoma 2 (AIM2)-like receptors (ALRs) are responsible for the recognition of DAMPs. Several members of NLRs and ALRs may bind to apoptosis-associated speck-like protein containing a caspase recruitment domain (CARD) (ASC) and initiate inflammasome formation. These intracellular molecular platforms, on the one hand, trigger proteolytic activation of different inflammatory caspases and cytokines, like caspase-1-dependent processing of IL-1β and IL-18 precursors, on the other hand, may promote pyroptosis [16]. Such assembly of inflammasomes basically requires two signals, at least in the case of the NLR family pyrin domain-containing protein (NLRP)3 inflammasome (Figure 1).

First, the priming signal via TLRs induces mostly NF-κB-mediated expression of inflammasome components and regulates their post-transcriptional modification. The second signal is provided by DAMPs to activate the assembly per se [17]. The huge array of DAMPs most probably indirectly impinges on the activation of inflammasomes through altering mitochondrial functions, ionic movements, phagosomal and plasmalemmal stability, and cell volume [16].

In the brain, the neurovascular unit (NVU) establishes an intimate structural and functional connection among microvascular endothelial cells, pericytes, glial cells, neurons, and extracellular matrix components. Primary functions of the NVU are the development and maintenance of the blood-brain barrier (BBB) and neurovascular coupling. Cerebral endothelial cells (CECs) continuously lining brain capillaries are armed by a fourfold defense line, a paracellular, a transcellular, and an enzymatic barrier, and efflux pumps. Due to these properties, oxygen and nutrients pass through the barrier and simultaneously, potentially noxious substances are kept out [18]. Located abluminally, pericytes contribute to maintaining the exceptional tightness of the BBB, control cerebral blood flow (CBF), clear the extracellular space from cellular debris and foreign molecules, and actively participate in angiogenesis [19]. With their endfeet forming the external surface of the capillary wall, astrocytes also play important role in the induction of brain endothelial barrier functions. Moreover, they provide an interface between the vessels and neurons, which allows them to respond dynamically to synaptic activity and metabolism of neurons and to regulate CBF [20]. Adjacent neurons send processes to a nearby capillary that courses ~8–15 µm from the closest neuron depending on the particular brain region [21,22]. In connection with astrocytes, neuronal activity provides the basis for functional hyperemia, during which increase in CBF is mediated by vascular cells [23].

Besides these previously mentioned neurovascular functions, cells of the NVU are also recognized for the role in the regulation of inflammation in the central nervous system (CNS) [24]. Inflammasome receptors appear to have a defined expression in cell types of the NVU with predominant expression of NLRP3 in endothelial cells; NLRP1-3, NLR family CARD domain-containing (NLRC)4 in pericytes; NLRP2 and NLRP3 in astrocytes; NLRP1, NLRP3 and AIM2 in neurons; and NLRP1, NLRP3 and NLRC4 in microglia [25,26,27]. Microglia are widely known to be in the front line of inflammatory responses in the CNS. As the topic is far too diverse and their role in neuroinflammation has recently been discussed in great detail elsewhere, the reader is kindly referred to those excellent reviews [28,29,30,31]. Here we mainly encompass the data on cells of the NVU other than microglia, videlicet endothelial cells, pericytes, astrocytes, and neurons. All of these cell types are known to express a diverse range of PRRs, other inflammasome components and cytokines with some preferential pattern [24]. Experimental data strongly indicate that brain vasculature is as much affected by inflammation as neural tissue. Growing body of literature supports the idea that the NVU takes center stage in age-related neurological diseases, and of this, inflammasomes are undoubtedly crucial mediators. In this review, the focus is placed on the link between inflammation and healthy brain aging. In addition, we aim to summarize the integral role of inflammasomes in aging-related diseases of the CNS with special emphasis on stroke and neurodegenerative disorders, including Alzheimer’s disease (AD), Parkinson’s disease (PD), and amyotrophic lateral sclerosis (ALS).

## 2. Healthy and Pathological Aging. Inflammaging

Normal aging of the human brain involves structural and functional alterations that commonly begin in midlife and are accompanied by a subtle and slow cognitive decline. This is associated with mild anatomical changes, including neuronal loss, glial proliferation in the cortex, loss of dendritic spines, and 2–3% decrease in brain volume per decade [32,33]. On the other hand, in pathological or premature aging of the brain, all these changes occur in a more intense and uncontrolled manner, with accelerated cognitive impairment and increased risk of dementia, neurodegeneration and stroke. Although differentiating abnormal from normal cognitive decline is often complicated, a few clinical symptoms are critical in this respect. In healthy aging, moderate decline in some cognitive abilities is considered to be acceptable (decrements in learning and attention, difficulty in understanding rapid speech, impaired word retrieval ability and defects in working and episodic memory), while autobiographical knowledge base and emotion processing remain relatively unchanged. In mild cognitive impairment, decline in mental abilities is apparent, but functional independence is still maintained. In major neurocognitive disorder or dementia (especially AD, which is the most common form of dementia), a patient’s cognitive decline already impairs independent living [34,35].

Among hallmarks of brain aging, dysregulated energy metabolism occupies center stage, located as an inner ring of the aging wheel [36]. This wheel is fueled by the nine other hallmarks, among which mitochondrial dysfunction, oxidative damage, impaired waste disposal mechanisms and inflammation are directly linked to inflammasome functions, as detailed below.

Aging is associated with increased activity of the innate immune system, during which TLR and inflammasome signaling are activated not only by pathogens but mainly by endogenous factors (including damaged, oxidized, and aggregated proteins) that accumulate with time [37,38]. Further sources that contribute to the increased inflammatory status in aging include immunosenescence, accumulation of cellular debris and senescent cells. These cells utilize inflammasomes to drive the senescence-associated secretory phenotype (SASP), which acts as a double-edged sword. On the one hand, it triggers clearance of senescent cells by the innate immune system. On the other hand, however, it also generates a pro-inflammatory milieu promoting inflammaging [39]. Damaged mitochondria are another major source of DAMPs, e.g., cardiolipin, ATP or mitochondrial DNA (mtDNA). In addition, mitochondria release reactive oxygen species (ROS) to induce oxidative stress, which triggers DAMP generation, while defective mitophagy leads again to hyperactivation of inflammatory signaling pathways [40,41]. As a result of DAMP accumulation and consequent inflammasome activation, mild chronic inflammation occurs and persists during aging.

Taken together, inflammation is a prominent aspect of normal brain aging, but it is also a key factor in the switch from the healthy phenotype to neurodegeneration [42]. This indeed raises the question of how to distinguish between healthy and pathological inflammaging. In line with the central role of maintained energy balance in bolstering brain health during aging [36], Akintola and van Heemst have approached this problem from the perspective of the equilibrium in metabolism and inflammation. In accelerated aging (for example in obesity as a model of pathological aging), insulin sensitivity tends to decrease, and chronic inflammation increases, while in delayed aging we see opposite tendencies [43]. Thus, healthy aging requires proper metabolism and communication among cells of the immune system in the brain and in the periphery, to maintain the balance between pro-inflammatory and anti-inflammatory factors [15]. Without chronic pathologies, aged individuals considered healthy show increased plasma levels of anti-inflammatory transforming growth factor β (TGFβ) and cortisol besides pro-inflammatory cytokines (like IL-18 and IL-6), providing partial evidence for this theory [14]. Endothelial cells and pericytes in the brain vasculature can detect all these serum factors, and can interpret the information to neurons and other cells in the parenchyma. If the fine balance tilts to the pro-inflammatory side, pathological inflammaging occurs.

The key role of inflammation in aging is supported by gene expression studies as well. Among the 36 genes differently expressed in human hippocampal brain tissue of old (age ≥ 60 years) compared to young (age ≤ 45 years) individuals, 90% were linked to inflammation or immune system activation [44]. In another microarray study, comparison of hippocampal samples from young (20–59 year-old) and old (59–99 year-old) people showed overexpression of several inflammation-related genes in old study participants, including cytokines (*IL6*, *IL1B*, *TNF*, *IL10*), modulators of cytokine signaling (*SOCS3* and *IRAK3*) and elements of TLR signaling (*TLR2*, *TLR4*, *TLR7*, *MYD88*) [45]. Gene expression alterations in different human brain regions were accentuated in males in comparison to females, which might be related to the poorly understood phenomenon that human longevity seems strongly influenced by gender [46].

Age-related structural and functional changes have been described in cells of the NVU as well [24,47]. Here we will focus only on inflammatory responses. In primates, intensified release of IL-6 was detected in isolated CECs from old (22 and >25 years old) individuals compared to young adults (3–8 years old) and neonates (3 days old) [48]. In aged mouse endothelial cells, elevated concentration of TNFα and reduced level of tight junction proteins with increased BBB permeability were observed [49]. At transcriptional level, a subgroup of cerebromicrovascular endothelial cells, 10.06% of the overall population, has recently been shown to have high senescence gene enrichment score in aged mouse brain, most of the upregulated genes being inflammation-related [50]. Likewise, a transcriptome analysis has evinced that CECs isolated from cortices and hippocampi of aged mouse have a pro-inflammatory and activated gene expression profile relative to CECs of young counterparts. Among proteins with vascular function associated with age-related changes, VCAM1, whose expression was elevated by circulating factors, most putatively by cytokines such as IL-1β and TNFα, was a telltale sign of microglial activation, impaired neurogenesis and defective cognition [51]. In the aging brain, astrocytes change their morphology from typically stellate shape with long, radial processes to short and stubby phenotype, and become reactive, which is attributed to an enhanced inflammatory state [33]. Age-related enrichment of IL-1β, IL-18, NF-κB and pro-caspase-1 was found in mouse astrocytes, which was partially dependent on activation of NLRP3 inflammasomes. Using NLRP3^−/−^ transgenic mice, the authors could decrease the extent of astrogliosis, the activation of inflammasomes and the age-related alterations in the transcriptome of old animals [52]. Overall, CECs, astrocytes and microglia, but not neurons, shift their gene expression pattern to an inflammatory profile, while synapse elimination and neuronal damage are secondary to glial activation [33,53]. On the other hand, NF-κB activation in hypothalamic neurons still seems to be associated with aging [54]. This is also very important because the hypothalamus is an essential center of metabolic and homeostatic control, and stress responsiveness, thus it emerges as a key structure for the neuro-immune axis in inflammaging [43].

Taken together, accumulation of DAMPs in the aging brain results in induction of inflammaging with several structural and functional consequences affecting vascular cells, glial cells and neurons. The balance between pro- and anti-inflammatory factors can easily be altered and metabolic dysfunction occurs in the brain, resulting in the development of neurodegeneration [55].

## 3. Neurovascular Inflammasome Activation in Alzheimer’s Disease (AD)

AD is a leading cause of dementia, currently affecting about 30–35 million people, with an increasing tendency of prevalence. AD is a chronic neurodegenerative disease with poorly understood causes and pathomechanism; a condition that progresses gradually towards a severe cognitive impairment and eventual death. Affected people—i.e., approximately 6% of the population over 65 years—increasingly rely on others for assistance and no currently available treatment has been clearly shown to delay the progression of the disease.

At the neuropathological level, AD is characterized by a deterioration of synapses, neuronal loss and brain atrophy, associated with accumulation of amyloid-β (Aβ) plaques, hyperphosphorylated tau protein aggregates and neurofibrillary tangles in the brain [56,57]. Neurovascular dysfunction, including decreased CBF, BBB disruption and impaired Aβ clearance, also contribute to the pathogenesis of the disease. Aβ can form deposits on cerebral vessels leading to cerebral amyloid angiopathy (CAA), which leads to compromised BBB integrity, microbleedings, vascular inflammation and leukocyte entry into the brain [58]. It is now clear that neurovascular inflammation contributes significantly to AD pathology. In this reaction, all cells of the NVU, mainly microglia, astrocytes, but neurons and vascular cells as well, and infiltrating cells take part.

Several studies have demonstrated the abundant number of reactive microglia surrounding Aβ plaques in AD [59,60]. Microglial phagocytosis of Aβ in senile plaques has been known to induce microglial activation and secretion of inflammatory cytokines such as IL-1β through activation of the NLRP3 inflammasome both in a double transgenic (APP695/PSEN1) mouse model of AD and in cultured microglia as well [61,62]. Cathepsin B release and endosomal rupture are also important mechanisms of NLRP3 inflammasome activation [62], and cathepsin B inhibitors could partially restore impaired memory and reduced Aβ plaque accumulations in a murine model [63]. Using APPswe/PS1dE9/NLRP3^−/−^ transgenic mice, the authors could prevent caspase-1 cleavage and decrease total IL-1β in brain samples, furthermore, animals were protected against memory deficit and amyloidogenesis [64]. Elevated levels of caspase-1, IL-1β, IL-6 and IL-18 have also been detected in the brain tissues of AD patients [56,65,66]. Notably, in addition to being secreted from microglia and infiltrated neutrophils, IL-1β can influence all the other cells of the NVU and trigger a vicious cytokine cycle, which induces additional (mostly pro-inflammatory) cytokine secretion and the overexpression of amyloid precursor protein (APP) [66,67].

As perivascular Aβ depositions also initiate activation of astrocytes, astrogliosis is a hallmark phenomenon in AD [68]. When activated, astrocytes release IL-1β [69] and their activation appears to last longer than that of microglia, outlining their role in neuroinflammatory responses [68]. The role of astrocytes in AD pathomechanism, however, remains controversial. On one hand, astrocytic IL-1β can enhance the production of APP and Aβ in neurons, contributing to further generation of Aβ depositions and neuronal cell death [70]. On the other hand, astrocytes can phagocytose Aβ and interestingly, this seems to be dependent on the presence of ASC, since ASC^+/−^ astrocytes display a higher phagocytic activity and release more C-C motif chemokine ligand (CCL)3 compared to ASC^+/+^ and ASC^−/−^ astrocytes [71]. Furthermore, exposure of rat astrocytes to 10 µM Aβ upregulated expression of IL-1β approximately ninefold above control level [72]. Since NLRP1, NLRP2 and NLRP3 inflammasomes are all present in astrocytes, IL-1β might be produced by either pathway [73,74].

In the McGill-R-Thy1-APP transgenic rat model of AD, IL-1β upregulation was exclusively detected in pyramidal neurons in the pre-plaque phase of AD, while in post-plaque phase IL-1β was elevated in glial cells as well [60]. This implicates the role of neuronal inflammasome activation in the early events of Alzheimer’s pathogenesis; however, limited information is available on this topic. As Aβ has also been reported intraneurally in AD [75], it seems likely that it might serve as a DAMP in neurons as well. In the APPswe/PS1dE9 mouse model of AD, neuronal NLRP1 activation was reported, while neuronal pyroptosis and cognitive impairments could be reduced with siRNA (small interfering RNA) to knockdown NLRP1 [76]. Furthermore, it has also been reported that primary human neurons express functional NLRP1, NLRC4, and AIM2, and serum deprivation induces NLRP1, caspase-1 and caspase-6 activation, along with ASC speck formation in vitro [77]. Besides NLRP1, neuronal NLRC4 activation might also contribute to AD pathogenesis. When exposed to palmitate-treated astrocyte conditioned media containing IL-1β and TNFα, hyperphosphorylation of tau and activation of NLRC4 inflammasome occurred in neurons [73].

Pericyte loss and degeneration have also been demonstrated in AD [78,79]. Since Aβ depositions are localized perivascularly, pericytes suffer prolonged exposure to Aβ causing cell death and detachment from blood vessels [80]. Brain pericytes express TLR4 and upon inflammatory stimuli they are able to increase production of ROS, nitric oxide (NO), IL-1β, IL-6, and TNFα [81,82]. Glial IL-1β release or ATP from damaged cells may further stimulate inflammatory responses in pericytes but their role in AD is still not yet fully understood.

Little is known about the role of endothelial inflammasome activation in AD. Exposure of cultured human CECs to Aβ_1–40_ elicited expression of inflammatory genes such as *CCL2*, *IL1B*, and *IL6*, which was also confirmed using brain tissues of AD patients [83]. Interestingly, isolated brain microvessels from AD patients released neurotoxic factors that killed neurons in vitro but the identity of these factors remained unclear [84]. Later it was shown that brain microvessels from AD individuals secreted high levels of NO, TGFβ1, TNFα, CCL2, IL-1β, IL-6, and IL-8 [85]. Importantly, inflammatory activation of CECs not only acts on brain cells, but promotes leukocyte diapedesis into the CNS, including T cells [86,87], and neutrophils [88], which further accelerate neuroinflammation and cognitive impairment.

These results suggest that all elements of the NVU can contribute to the exaggeration of inflammatory mechanisms in AD and inflammasome activation might act as an acceleratory mechanism in the propagation of the disease.

## 4. Neuroinflammatory Changes in Parkinson’s Disease (PD)

PD is a progressive extrapyramidal movement disorder caused by degeneration of dopaminergic neurons in the substantia nigra pars compacta. The disease has its onset usually after the age of 60, and starts with motor symptoms of extrapyramidal origin (a clinical syndrome called parkinsonism, composed of bradykinesia, muscle rigidity, tremor, gait and balance problems, etc.), but non-motor symptoms (depression, anxiety, sleeping problems, and cognitive impairment) also appear over the years. Degeneration of dopaminergic neurons is accompanied by accumulation of α-synuclein aggregates in Lewy bodies and Lewy neurites of affected neurons. Tau aggregation in neurofibrillary tangles has also been described [89]. As recently shown, amyloid fibrils of α-synuclein, having β-sheet secondary structure, may propagate within the brain in a prion-like manner [90]. As a progression of PD, memory and thinking problems may arise and worsen over time, leading to dementia. If dementia precedes parkinsonism, the disease is called dementia with Lewy bodies [91]. In fact, toxic α-synuclein oligomers are found in different neurons and glial cells in a collection of diseases called Lewy body disorders [92]. Being the most well-known among these, only PD will be referred to in this chapter.

In the context of disease pathomechanism, neuroinflammatory changes have been increasingly recognized as key elements in the degeneration of neurons [93]. Main cellular players in this phenomenon are microglia and infiltrating T cells [94], which activate the pro-inflammatory machinery, including inflammasomes. Besides general neurodegeneration-related mechanisms, that have been connected to inflammasome activation (including mitochondrial dysfunction, mitophagy, and inflammatory cytokines) (reviewed by [95,96]), α-synuclein might also act as a central element in inflammasome activation in PD. Released by degenerating neurons, α-synuclein acts as a DAMP and primarily activates microglial cells [97,98]. A number of studies have verified that microglial NLRP3 inflammasome is activated in human post-mortem tissue of PD patients and in multiple experimental mouse models of PD [99,100], along with increased ASC, caspase-1, and IL-1β levels. However, another study reported increased NLRP3 levels in the mesencephalic neurons of PD patients on post-mortem tissue and identified dopaminergic neurons as cells-of-origin of inflammasome activation in PD [101]. The authors have also identified a rare *NLRP3* polymorphism associated with decreased risk of PD.

In the α-synuclein pre-formed fibril (PFF) mouse model of PD, inhibition of NLRP3 activation with MCC950 not only reduced inflammasome activation, dopaminergic neuronal loss, and motor deficits, but also prevented α-synuclein aggregation [99]. The exact molecular mechanism behind this effect is not fully understood, but it further highlights the role of NLRP3 in the pathogenesis of PD, since it suggests a back and forth amplification pathway between α-synuclein pathology and NLRP3 activation.

Modulation of NLRP3 activation with miR-7 [100], the peroxisome proliferator-activated receptor (PPAR) agonist GW501516 [102], or caspase-1 inhibition [103] have all been reported to alleviate dopaminergic degeneration in PD mouse models, providing possible novel therapeutic targets. Furthermore, the hepatic inhibition of NLRP3 was also shown to attenuate dopaminergic neurodegeneration in the 1-methyl-4-phenyl-1,2,3,6-tetrahydropyridine (MPTP) mouse model of PD, outlining the role of systemic activation of NLRP3 in PD. This is further supported by reports of elevated NLRP3, caspase-1 and IL-1β in the blood serum of PD patients and its correlation with α-synuclein [104,105].

TLR2 was also detected in the post-mortem tissue of PD patients in microglia and dopaminergic neurons, and was shown to correlate with α-synuclein accumulation [106]. However, the authors also reported that neuronal, rather than glial TLR2 expression increased in association with disease staging. This observation might shed some light on the previously mentioned differences in the reported cell-type specificity of inflammasomes in PD, as disease progression and the experimental models might have a drastic impact on the extent and specificity of the observed inflammasome/PRR activation.

Additionally, TLR4^−/−^ mice were also reported to exhibit reduced neuroinflammation, less α-synuclein abnormalities and decreased dopaminergic neuronal loss in the MPTP mouse model of PD [107,108].

The evidence listed here suggests a complex interaction network between inflammasome activation, α-synuclein accumulation and degeneration of dopaminergic neurons. However, the specific molecular and cellular pathways in this network and the possible involvement of additional inflammasomes are still not fully understood and require further elaboration. On the other hand, as the impact of certain inflammasome components has already been verified by KO transgenic mice or pharmacological inhibition in animal models of PD, evaluation of inflammasomes as potential therapeutic targets in PD seems increasingly reasonable.

## 5. Amyotrophic Lateral Sclerosis (ALS): Role of Inflammasomes

ALS is the most common motor neuron disease, characterized by the degeneration of upper and lower motor neurons in the motor cortex, brainstem, and spinal cord [109]. ALS is considered to be a relentlessly progressive disorder with very poor prognosis, as most patients die in less than 5 years after diagnosis, typically from respiratory failure due to the extensive muscle weakness.

While a number of factors have been proposed to contribute to disease progression, family history, male sex and aging are considered to be the major risk factors of ALS [110,111]. Aging in ALS is not only of epidemiological, but also of cellular importance, as neurons are post-mitotic cells and the vulnerability of motoneurons is known to increase in an age-related manner [112]. Despite the primary involvement of motor neurons, astrocytes [113], microglia [114], and infiltrating immune cells [115] have also been implicated in the underlying pathomechanism of the disease. Besides being recognized as a non-cell autonomous disease, the significant role of neuroinflammation in the disease progression [116], and early changes in astrocytic gene expression [117] suggest that neuroinflammation may precede motoneuronal loss pre-symptomatically. Moreover, oxidative stress and mitochondrial dysfunction are well-described factors in the pathogenesis of ALS [118], both of which are known to induce inflammasome activation [119].

Indeed, NLRP3 inflammasome was confirmed to be activated in the superoxide dismutase (SOD)1(G93A) transgenic model and in human sporadic ALS patients as well [120]. Notably, NLRP3, ASC, caspase-1, and mature IL-1β could already be detected at a pre-symptomatic stage in SOD1 mice, predominantly in spinal cord astrocytes. Increased level of NLRP3 was also confirmed in the post-mortem spinal cord tissue of ALS patients. However, western blot analysis only found ASC, active caspase-1, and IL-18 levels to be significantly increased in human ALS tissue. While the lack of increase in mature IL-1β levels might be unexpected, it is in line with previous observations showing that among IL-1 family cytokines, only IL-18 was upregulated in sera collected from ALS patients [121]. Of note, besides IL-1β, IL-18 is also an inflammasome-activated cytokine.

In SOD1 mice, degeneration of the anterodorsal thalamic nucleus (ADT) in the symptomatic stage was accompanied by activation of NLRP3 in ADT neurons and astrocytes, along with increased neuronal IL-1β levels [122]. This cell-type specificity of NLRP3 activation is somewhat contrasting compared to the previous study of the authors [120], where they reported mostly astrocytic NLRP3 elevation in the spinal cord of the same transgenic model. In fact, this difference in neuronal vs. astrocytic NLRP3 elevation might emerge from the regional differences of NLRP3 expression, priming and activation.

Interestingly, administration of 17β-estradiol was shown to reduce NLRP3 activation and IL-1β levels in SOD1 mice, along with the reduction of motoneuronal loss and increased survival [123]. Whether this is a direct effect of 17β-estradiol on NLRP3 inflammasome assembly, or the indirect consequence of reduced motoneuronal degeneration, is not yet fully understood. NLRP3 activation has also been reported in the brainstem of SOD1 mouse, along with increased TLR4 expression [124], highlighting the fact that other PRRs might also play a role in the progression of the disease. On the other hand, administration of the TLR4 inhibitor TAK-242 only showed moderate effects on delaying disease progression and motoneuronal loss, without affecting survival of SOD1 mice [125].

It is also worth mentioning that even though several papers have reported the lack of NLRP3 overexpression in microglia in ALS [120,122], in other ALS studies, NLRP3 was shown to be activated in microglia [126,127,128]. It should also be pointed out that activation of microglial NLRP3 inflammasome with TAR DNA-binding protein 43 (TDP43)—the major pathological protein in sporadic ALS and the closely related frontotemporal dementia—was shown to be toxic to motoneurons in a microglia-motoneuron co-culture [128]. Thus, even if not directly acting on motoneuronal NLRP3, TDP43 might fuel further neuronal loss and inflammation in the NVU.

These results highlight the role of inflammasome activation in the progression of ALS pathology and raise the possibility of identifying inflammasomes as points of intervention. Although, similarly to AD and PD, most of the studies focus on the involvement of NLRP3 in ALS too, it is reasonable to assume that additional inflammasomes might also contribute to the evolution of neuroinflammatory mechanisms. These questions, along with the specific origin of inflammasome activation and the possible inflammasome-mediated interactions between cells of the NVU, however, are to be studied and discussed in future reports.

## 6. Neurovascular Inflammaging in Stroke

Ranked as the second dominant cause of mortality worldwide, stroke mostly occurs in the aged population [129,130], with twofold increase in incidence every decade over the age of 55 [131]. Based on pathology and clinical features, stroke is generally divided into ischemic (responsible for about 80% of all strokes) and hemorrhagic types [132]. During ischemic stroke, a brain region undergoes an abrupt cessation of blood flow, most commonly because of a thrombotic or embolic occlusion in the middle cerebral artery (MCA) territory [133,134]. Subsequent reperfusion, unfortunately, not only minimizes the effects of the hypoxic insult, but may paradoxically enhance the injury by ROS-mediated mechanisms [135]. In contrast to ischemia, hemorrhagic stroke occurs when blood accumulates in either the subarachnoid space or in the brain parenchyma as a consequence of a ruptured vessel [136].

A set of cellular and molecular events, including metabolic imbalance, excitotoxicity, oxidative stress, BBB dysfunction and secretion of pro-inflammatory cytokines from cells of peripheral and resident origin lead to differential cell death depending on temporospatial features of the injury [133,136,137]. By virtue of being the most vulnerable, neurons liberate DAMPs once they are damaged or engaged in cell death [133]. These endogenous danger signals, in turn, alert PRRs expressed both in peripheral immune cells and cells of the NVU [27,137]. Notably, activation of these PRRs—some of which are known to form inflammasomes—might be regulated post-stroke in response to a diverse range of self-molecules, including ROS, ATP, HMGB1, certain heat shock proteins (HSPs), histones, genomic and mitochondrial DNA [138,139,140,141,142,143,144]. All these stimuli facilitate production and secretion of IL-1β and IL-18, ultimately leading to further neuronal and glial death in the ischemic and hemorrhagic tissue, and contributing to exacerbation of neurological deficits [139,145,146]. Even though majority of the studies are centered around the NLRP3 inflammasome and its role in stroke pathophysiology [138,147], multiple other inflammasome constituents have been addressed in inflammatory responses of the NVU related to stroke. These are NLRP1 [139,148], NLRP2 [149,150], NLRP6 [151], NLRP12 [152], NLRC4 [145,153], and AIM2 [153], as detailed below.

Apart from neuronal expression, NLRP1 inflammasome has also been found in astrocytes and microglia/macrophages with a complex spatial and temporal expression pattern in the mouse brain after stroke [148]. Comparable results showed an elevated expression of NLRP3 inflammasome components and downstream effector targets in conjunction with NLRP1 in mouse and human brain tissue compromised by stroke [139]. Regarding the distribution of individual inflammasome components in the penumbra post-stroke, NLRP1 and NLRP3 were determined to be predominantly neuron-specific, whereas ASC showed a disproportionate expression with microglia being the major ASC-expressing cell type, but few neurons and astrocytes also expressed it [148,154]. Intriguingly, NLRP3 expression was first downregulated, then returned to basic levels over time in the peri-infarct zone [154]. Previous observations concerning the implication of NLRP3 in stroke were challenged by Denes et al., proposing that NLRP3 inflammasome activation is dispensable in the acute phase of stroke. Instead, NLRC4 and AIM2 along with ASC were suggested to be the major contributors to brain injury in the MCA occlusion (MCAO) model of stroke in mice [153], as further evidenced by others as well [145]. In concordance with the findings of Denes et al. [153], an independent study using photothrombotic model of stroke reported that NLRC4 and AIM2, but not NLRP3, play a key role in a primary stroke incident. Vice versa, expression of NLRP3, but not those of NLRC4 and AIM2, was markedly increased in response to recurrent stroke evoked in the contralateral cortex, probably due to the antecedent ischemic stroke as a priming step for NLRP3 inflammasome activation. Moreover, extracellular ASC specks, which might originate from the first stroke-affected area, were shown to contribute to the aggravation of recurrent stroke in NLRP3-dependent manner [155]. Recently, the NLRP6 inflammasome pathway has been acknowledged to be involved both in in vitro and in vivo models of ischemic stroke, functioning mainly in astrocytes and decreasing the viability of neurons [151,156]. Also, a new study showed that NLRP10, the only member of the NLR family without LRR domain and exhibiting a dual role in inflammation, participated in the progression of inflammation by inducing NLRP12 inflammasome activation which could be hindered by deleting the *NLRP10* gene [152].

Among relevant inflammasome activators in stroke, extracellular ATP is one of the most important. After cerebral ischemia, initially high extracellular levels of ATP, originated from injured cells, can enhance NLRP3 inflammasome activation [147,157]. On the other hand, increasing cellular levels of AMP or, to some extent, depletion of ATP, cause the transition of the common energy sensor AMP-activated protein kinase (AMPK) into the active state [158]. AMPK has recently emerged as a repressor of NLRP3 activation in aging [159]. There is a substantial debate on the role of AMPK in neuronal salvage, either detrimental or protective [158]. Using oxygen and glucose deprivation (OGD) and MCAO in rodent cell/tissue culture and brain, respectively, certain herbal agents were found to exert anti-inflammatory and neuroprotective effects on neurons and glial cells by suppressing NLRP3 inflammasome activation through activation of the AMPK pathway [160,161], activation of autophagy [162] and via upregulating the NAD-dependent deacetylase sirtuin-1 [163]. Similarly, it was shown that a BBB-permeable recombinant adiponectin peptide (APNp) could reverse oxidative, inflammatory and apoptotic processes by inhibiting NLRP3 inflammasomes in murine astrocytes via AMPK signaling [164]. Also of note, ATP has been proven to activate the NLRP2 inflammasome in a purinergic receptor-dependent pathway in cultured human astrocytes, giving rise to a fivefold and twofold increase in the production of IL-1β and IL-18, respectively, compared to the control state [165]. In a later study, basal expression of NLRP2 was confirmed in mouse astrocytes, mainly in the hippocampus, and its protein level was markedly elevated in ischemic conditions [149]. Furthermore, in a recent paper, apoptosis signal-regulating kinase 1 (ASK1)—which is required for the induction of ATP-dependent apoptosis [166]—was found to induce astrocytic NLRP2 inflammasome activation in the OGD and MCAO models [150]. Overall, these data suggest that ATP can be a major ”kindling” factor of various signaling pathways, all of which converge to inflammasome activation followed by release of cytokines, which may escalate inflammation in the stroke-damaged brain. In this context, the NLRP2 inflammasome seems to be a relatively new player with a function not yet fully elucidated.

Other potent inflammasome activators in stroke are ROS molecules. In relation to the metabolic disturbance in the ischemic phase, and as a result of the reoxygenation injury, a surfeit of ROS is generated by many enzymes in the brain owing to intrinsic pro-oxidant features that make it prone to be damaged shortly following ischemia. Typical ROS include superoxide, hydroxyl radical, NO, peroxynitrite, and hydrogen peroxide (H_2_O_2_), which have been shown to be crucial mediators of oxidative stress that aggravates tissue injury in stroke [167]. ROS are mainly produced by perturbation of the mitochondrial electron transport chain and mitophagy [138,168], and the excessive amount of ROS is thought to trigger activation of the NLRP3 inflammasome through thioredoxin-interacting protein (TXNIP) [169]. Indeed, it has been demonstrated that oxidative stress-related TXNIP/NLRP3 inflammasome activation is inhibited by curcumin, the main natural polyphenol of *Curcuma* species, in neuronal cells [160]. As a different approach, idebenone—a synthethic antioxidant and anti-aging compound—has recently been proposed to counteract the NLRP3 inflammasome-activating effects of ROS and mtDNA in neuron and microglia co-culture in response to OGD [170]. Vascular cells may also be damaged by ROS. A recent study showed that H_2_O_2_ regulated a number of cellular mechanisms in brain microvascular endothelial cells in a concentration-dependent manner, thereby altering the BBB [171]; however, either H_2_O_2_ or the ROS donor 2,3-dimethoxy-1,4-naphthoquinone (DMNQ) failed to change the expression of several stroke-related inflammasomes in CECs [25]. In another study, ruscogenin, a steroidal saponin, significantly reduced NLRP3 and TXNIP expression and formation of ROS in CECs, hence enhancing barrier functions [172].

In the NVU, pericytes have been described to have a putatively indispensable role in diverse functions associated with ROS in ischemic stroke [173]. In particular, pericytes appeared to cause a prolonged constriction of microvessels after ischemia in mice that was induced by oxidative stress. On the contrary, pericyte-induced microvascular obstruction could be reversed by application of the free radical scavenger N-tert-butyl-α-phenylnitrone (PBN) and the nitric oxide synthase (NOS) inhibitor Nω-nitro-L-arginine (L-NNA) before reperfusion [174]. Recently, it has been shown that human brain vascular pericytes (HBVPs) rapidly (i.e., in 2 h) tend to contract in response to iodoacetate and antimycin-A, agents that mimic oxidative conditions in ischemic stroke [175]. Inflammatory mechanisms may also be activated in pericytes, e.g., Nyúl-Tóth et al. found that H_2_O_2_ caused NLRP3 upregulation in HBVPs [27]. Besides mitochondria, NADPH oxidase (NOX) is another elementary source of ROS in stroke [168]. In this enzyme family, NOX4 is upregulated in pericytes in the penumbra after acute brain ischemia, increasing production of matrix metalloproteinase-9 (MMP-9) [176], that induces plasma leakage focally at pericyte somata [177]. Moreover, in a hemorrhagic model of stroke in mice, IL-1β was supposed to mediate MMP-9 activation that could be reduced by the caspase-1 inhibitor Ac-YVAD-CMK [178]. In support of this assumption, IL-1β released from endothelial cells in an NLRP3-dependent manner could amplify the inflammation locally with other pro-inflammatory cytokines, resulting in the upregulation of MMP-2/-9 and subsequent BBB breakdown [179,180,181]. These data altogether suggest that ROS and pro-inflammatory cytokines play a pivotal role in mediating expression and activity of the gelatinase subgroup of MMPs derived from different sources of cells of the NVU, which may be controlled directly and/or indirectly by inflammasomes [179,182,183].

In vitro studies have reported that NLRP3 inflammasome is activated by cell volume changes too [184,185]. Ischemia-reperfusion injury is commonly accompanied by cell swelling that causes cellular damage [179,186,187]. In fact, biophysical properties of the BBB are altered with perivascular edema, vacuolation and membrane damage and over time cytotoxic edema transforms into vasogenic edema and hemorrhagic stroke [179,187]. During cell swelling, aquaporin-4 (AQP4) specifically facilitates water uptake of perivascular astrocyte endfeet ensued by the compression of adjacent capillary lumen. When exposed to swelling, astrocytes show a nearly ninefold increase in the expression of *IL1B*, which can be diminished by interfering with the action of ATP on different targets [185]. This observation was underpinned by a recent finding that the NLRP3 inflammasome inhibition with MCC950 regulated the expression and distribution of AQP4 and alleviated the disruption of the BBB after MCAO in the infarcted brain region of mice [188]. Furthermore, NLRP3 deficiency was found to be beneficial as it could improve the neurovascular damage after stroke in mice [179].

The data detailed in this section clearly suggest that a complex, multicellular inflammasome activity gives rise to the aggravation of tissue injury and neurodeficits in the acute phase of stroke. Although not discussed here, such damage also results from the activity of infiltrating and perivascular immune cells apart from that of CNS-resident cells [189,190]. Improvement in understanding the role of multiple inflammasomes, their triggers and effectors in the development of the destructive, pro-inflammatory environment, provides potential for novel therapeutic approaches for the treatment of inflammation following stroke.

## 7. Conclusions

Aging is inadvertently linked to a chronic, low-grade, sterile inflammation (i.e., inflammaging) and is a leading risk factor for many neuroinflammatory diseases of the CNS, including AD, PD, ALS, and stroke. Mounting evidence indicates that inflammasomes, which are known to be expressed in cells of the NVU, play a determinant role in the aging process, and in the onset and progression of age-related diseases of the CNS. Literature data concerning expression of inflammasome proteins and effectors in cells of the NVU in age-associated CNS diseases are summarized in (Table 1).

**Figure 2:**
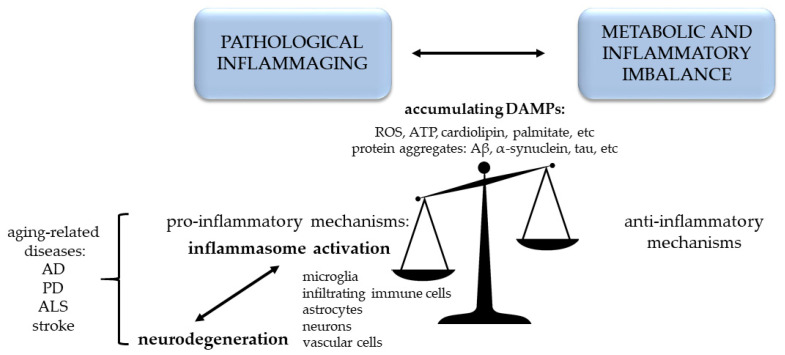
Pro-inflammatory shift in pathological aging. Aβ: amyloid-β, AD: Alzheimer’s disease, ALS: amyotrophic lateral sclerosis, ATP: adenosine triphosphate, PD: Parkinson’s disease, ROS: reactive oxygen species.

Both in health and disease, in cells that have already been primed by cytokines, DAMPs or hormones [191], inflammasomes can be activated in response to a wide range of host-derived by-products that are accumulated intra- and extracellularly with aging. Inflammasome activation, in return, leads to the release of pro-inflammatory cytokines (IL-1β or IL-18) and pyroptotic cell death, which eventually amplify the inflammation on site (Figure 2).

Thereby, the aging process is accelerated, and exacerbation of the disease occurs, if detrimental effects of inflammasome activation are not counterbalanced. The diseases reviewed here share common hallmarks associated with the damage to the NVU, namely neuronal loss and BBB disruption [192,193]. The cellular network of the NVU contributes to the inflammation as a whole through a combined reaction of inflammasomes that is partly determined by spatiotemporal expression pattern of inflammasome proteins in different cell types of the NVU. Indeed, with disease development, neurons become dysfunctional and die, releasing DAMPs that activate inflammasomes in neurons, glial and vascular cells. Such activation of inflammasomes drives further cell death. Conversely, silencing or knock-out of particular inflammasome protein-coding genes and using pharmacological compounds, which negatively regulate inflammasome-activating mechanisms or directly block inflammasome components or substrates, improve the detrimental consequences of the inflammasome activation in a defined time window.

This implies that targeting upstream activation of inflammasomes and downstream consequences of their activation in a specific period of time, is of critical importance in the therapy of age-related diseases. Most of the studies have explored the role of different inflammasomes in neurons and glial cells in neurodegenerative disorders and stroke, especially revolving around NLRP3 inflammasome, while inflammasomes of vascular cells such as endothelial cells and pericytes have not received considerable attention. It depends on future studies to provide valuable insights into the role of inflammasomes in these cells and elucidate further details on effects of inflammasomes with already recognized implication in aging and aging-associated CNS pathologies.

## Figures and Tables

**Table 1:** Changes in levels of inflammasome components and effectors in cells of the NVU in age-related diseases.

Disease	Cell Type	Experimental System	Inflammasome Components/Effectors	Reference
AD	astrocytes	McGill-R-Thy1-APP transgenic mouse	IL-1β↑	[60]
		primary mouse astrocytes treated with fibrillary Aβ	ASC↑, IL-1β↑	[71]
		primary mouse astrocytes treated with fibrillary Aβ	caspase-1↑, IL-1β↑	[74]
		primary rat astrocytes treated with palmitate	NLRC4↑, ASC↑, caspase-1↑, IL-1β↑	[73]
	neurons	McGill-R-Thy1-APP transgenic mouse	IL-1β↑	[60]
		APP/PS1 transgenic mouse	NLRP1↑	[76]
		primary human serum-deprived neurons	NLRP1↑, ASC↑, caspase-1↑	[77]
		primary rat neurons treated with Aβ	NLRP1↑, caspase-1↑, IL-1β↑	[76]
	microglia	APP/PS1 transgenic mouse	ASC↑	[64]
		mouse primary microglia or microglia cell line treated with fibrillary Aβ	ASC↑, caspase-1↑, IL-1β↑	[62]
PD	neurons	human mesencephalic post-mortem tissue from PD patient	NLRP3↑, caspase-1↑	[101]
		human mesencephalic neuron-derived cells treated with nigericin and LPS	NLRP3↑, caspase-1↑	[101]
	microglia	human substantia nigra post-mortem tissue from PD patient	NLRP3↑, ASC↑	[99]
		α-Syn(A53T) transgenic mouse	NLRP3↑, caspase-1↑, IL-1β↑	[100]
		6-OHDA mouse model	NLRP3↑, ASC↑, caspase-1↑	[99]
		MitoPark transgenic mouse	NLRP3↑, ASC↑, caspase-1↑	[99]
		PFF mouse model	NLRP3↑, ASC↑, caspase-1↑	[99]
		BV-2 cells treated with α-Syn	NLRP3↑, caspase-1↑	[100]
ALS	astrocytes	human post-mortem tissue (spinal cord)	NLRP3↑, ASC↑, caspase-1↑, IL-18↑	[120]
		SOD1(G93A) transgenic mouse (ADT)	NLRP3↑, ASC↑, IL-1β↑	[122]
		SOD1(G93A) transgenic mouse (spinal cord)	NLRP3↑, ASC↑, caspase-1↑, IL-1β↑, IL-18↑	[120]
		SOD1(G93A) transgenic mouse (spinal cord)	NLRP3↑, ASC↑, caspase-1↑, IL-1β↑	[126]
	neurons	SOD1(G93A) transgenic mouse (ADT)	NLRP3↑, ASC↑, IL-1β↑	[122]
	microglia	SOD1(G93A) transgenic mouse (ADT)	ASC↑	[122]
		SOD1(G93A) transgenic mouse (spinal cord)	ASC↑	[120]
		SOD1(G93A) transgenic mouse (spinal cord)	NLRP3↑, ASC↑, caspase-1↑, IL-1β↑	[126]
		primary mouse microglia treated with SOD1(G93A)	ASC↑, caspase-1↑, IL-1β↑	[126]
		primary mouse microglia treated with WT TDP43 or TDP43(A315T, Q331K)	IL-1β↑	[126]
		primary mouse microglia treated with WT TDP43 or TDP43(M337V)	NLRP3↑, IL-1β↑	[128]
Stroke	endothelial cells	MCAO mouse model	NLRP3↑	[179]
		OGD	NLRP3↑, caspase-1↑, IL-1β↑	[172,179]
		OGD	IL-18↑	[179]
	astrocytes	thromboembolic (CCAT) stroke mouse model	NLRP1↑, ASC↑, caspase-1↑	[148]
		MCAO mouse model	caspase-1↑	[150]
		MCAO rat model	NLRP3↑, ASC↑	[154]
		OGD	NLRP2↑	[149]
		OGD	NLRP3↑	[164]
		OGD	NLRP3↑, caspase-1↑, IL-1β↑	[161]
		OGD	NLRP6↑	[151]
		OGD	ASC↑, caspase-1↑, IL-1β↑, IL-18↑	[151,164]
	neurons	human stroke patient brain tissue	NLRP1↑, NLRP3↑, ASC↑, caspase-1↑, IL-1β↑, IL-18↑	[139]
		thromboembolic (CCAT) stroke mouse model	NLRP1↑, ASC↑, caspase-1↑	[148]
		MCAO mouse model	NLRP1↑, ASC↑, caspase-1↑, caspase-11↑, IL-1β↑	[139]
		MCAO rat model	NLRP3↑, ASC↑	[154]
		GD or OGD or IR simulation	NLRP1↑, NLRP3↑, ASC↑, XIAP↑, caspase-1↑, caspase-11↑, IL-1β↑, IL-18↑	[139]
		OGD (cultured with microglia)	NLRP3↑, caspase-1↑, IL-1β↑, IL-18↑	[170]
		OGD	NLRP6↑	[151]
	microglia	thromboembolic (CCAT) stroke mouse model	NLRP1↑, ASC↑, caspase-1↑	[148]
		photothrombotic stroke mouse model	ASC↑	[155]
		MCAO mouse model	NLRP3↑	[163,179]
		MCAO mouse model	NLRC4↑, caspase-1↑	[145]
		MCAO mouse model	IL-1β↑	[145,153]
		MCAO rat model	ASC↑	[154]
		MCAO rat model	NLRP3↑, caspase-1↑	[170]
		OGD	NLRP1↓, NLRC4↑, NAIP↓, AIM2↓, ASC↑, XIAP↓, caspase-11↑	[145]
		OGD	NLRP3↑	[145,179]
		OGD	NLRP6↑	[151]
		OGD	caspase-1↑, IL-1β↑	[145,179]
		OGD	IL-18↑	[179]
		OGD	NLRP3↑, caspase-1↑, IL-1β↑	[161]
		OGD	NLRP3↑, ASC↑, caspase-1↑, IL-1β↑	[162]
		OGD	NLRP3↑, caspase-1↑, IL-1β↑, IL-18↑	[170]

Aβ: amyloid-β, AD: Alzheimer’s disease, ADT: anterodorsal thalamic nucleus, AIM2: absent in melanoma 2, ALS: amyotrophic lateral sclerosis, APP: amyloid precursor protein, ASC: apoptosis-associated speck-like protein containing a caspase recruitment domain, α-Syn: α-synuclein , CCAT: common carotid artery thrombosis, GD: glucose deprivation, IL: interleukin, IR: ischemia-reperfusion, LPS: lipopolysaccharide, MCAO: middle cerebral artery occlusion, NAIP: nucleotide-binding oligomerization domain-like receptor family apoptosis inhibitory protein, NLRC: nucleotide-binding oligomerization domain-like receptor family caspase recruitment domain-containing, NLRP: nucleotide-binding oligomerization domain-like receptor family pyrin domain-containing protein, OGD: oxygen and glucose deprivation, 6-OHDA: 6-hydroxydopamine, PD: Parkinson’s disease, PFF: pre-formed fibril, PS1: presenilin-1, SOD1: superoxide dismutase 1, TDP43: TAR DNA-binding protein 43, XIAP: X-linked inhibitor of apoptosis protein, WT: wild type, ↑: increase, ↓: decrease.
